# Building the Next Bioethics Commission

**DOI:** 10.1002/hast.710

**Published:** 2017-05-22

**Authors:** Alexander M. Capron

## Abstract

*At every moment, somewhere in the world, a group of men and women are sitting around a table deliberating about an ethical issue posed by medicine and research, whether as a research ethics committee; a hospital or clinical ethics committee; a stem‐cell review committee; a gene transfer research committee; a biobank ethics committee; an ethics advisory committee for a medical or nursing association or nongovernmental organization; a state, provincial, national, or intergovernmental bioethics committee; or an ad hoc panel examining a particular development or case. However, the last national committee in the United States, the Presidential Commission for the Study of Bioethical Issues, held its final meeting at the end of August 2016 and closed its doors. Should we regret its departure? I believe that the United States would benefit from having another national bioethics advisory body, but I do not think that the commission should simply have continued under a new president in the same form. Instead, looking at the experience of that commission and its six predecessors—who they were, how they worked, the functions they served, and the problems they experienced—we can derive some useful ideas for anyone planning to build the next commission*.

## Comparative Analyses

The sun never sets on bioethics committees. At every moment, somewhere in the world, a group of men and women are sitting around a table deliberating about an ethical issue posed by medicine and research, whether as a research ethics committee (called an “institutional review board” in the United States); a hospital or clinical ethics committee; a stem‐cell review committee; a gene transfer research committee; a biobank ethics committee; an ethics advisory committee for a medical or nursing association or nongovernmental organization; a state, provincial, national, or intergovernmental bioethics committee; or an ad hoc panel examining a particular development or case.

Nonetheless, the sun has set on one such committee, the Presidential Commission for the Study of Bioethical Issues. Created by an executive order signed by President Obama on November 24, 2009, and launched into operation in the spring of 2010 once its full complement of thirteen members was appointed, this commission held its final meeting at the end of August 2016 and closed its doors. Should we regret its departure?

I believe that the United States would benefit from having another national bioethics advisory body, but I do not think that the commission should simply have continued under a new president in the same form. Instead, looking at the experience of that commission and its six predecessors (see the table)—who they were, how they worked, the functions they served, and the problems they experienced—we can derive some useful ideas for anyone planning to build the next commission.

## Who Should Be the Members?

The first commission established to examine the new category of issues raised by biology and medicine was the National Commission for the Protection of Human Subjects of Biomedical and Behavioral Research, which was authorized in the National Research Act of 1974 and appointed by the secretary of Health, Education, and Welfare. The impetus for the National Commission is well known: in 1972, a whistleblower's revelation of the forty‐year‐long U.S. Public Health Service observational study of untreated syphilis in poor African American men in rural Alabama caused public outrage and prompted Congress to act.

But the National Commission was not the first body appointed by the secretary of HEW, Caspar W. Weinberger, in response to those revelations; two years earlier, he had established the Tuskegee Syphilis Study Ad Hoc Advisory Panel. That body reached conclusions not only about the syphilis study but also about what the federal government should do to avoid a repetition of the study's mistakes. So the creation of the National Commission tells us at least three things. First, the representatives and senators did not believe that the problems revealed by the Tuskegee study would be dealt with adequately as an internal matter by the Department of HEW. Second, they wanted a broader range of the research ethics issues (not just problems in the Tuskegee study) to be publicly examined. And third, they took ownership of the issues by instructing the National Commission to report back not just to the executive branch but also to Congress.

This shaped part of the “who” of the first commission in ways that have largely continued for more than four decades. The eleven commissioners were drawn from a wide variety of fields but were not expected to serve as representatives with actual constituencies.^1^ Although George Annas complained that the block of five from “biomedical or behavioral research involving human subjects” would dominate the deliberations, the commission did not hew to the professional orthodoxy of the time (medical paternalism, for instance), although it agreed with the choice made by Congress, which had placed oversight of research projects with institutional review boards, a majority of whose members are typically researchers.^2^

The National Research Act permitted federal employees to be appointed as commissioners, but none were, and the charters of subsequent bioethics commissions—up until the latest presidential commission—have excluded federal employees from membership. While the members of the National Commission were appointed for the duration of that time‐limited body, the eleven members of its successor—the President's Commission for the Study of Ethical Problems in Medicine and Biomedical and Behavioral Research (President's Commission), which was expected to remain in place across presidential administrations—were appointed to four‐year terms staggered by having four of the original commissioners appointed for three years and three for two years.^3^ Although turnover can be disruptive, since new commissioners need to adapt to the way the body deliberates and join at a time when a number of reports are in mid‐preparation, it is nevertheless valuable. First, it links the commission to the current administration. Second, it provides an early test of the persuasiveness of the commission's draft reports: can they gain the endorsement of the new members?

Finally, with the professionalization of the field, some people have suggested that commissions should include more bioethicists. I believe that expertise (be it in bioethics, science, law, or whatever) is needed among the staff, consultants, and witnesses, but the commissioners ought to be a diverse group of thoughtful people who are capable of deliberating about, and reaching conclusions on, the issues in a way that is understandable to and responsive to the needs of the public. A commission's reports need to be intellectually respectable, but the primary audience for commissions is not one of moral philosophers or other academic experts.

## How Should They Work?

New staff members and commissioners are often leery of one basic rule that applies to bioethics commissions as federal advisory bodies: they must “do ethics in public,” as Albert R. Jonsen, a member of both the National Commission and the President's Commission, remarked.^4^ Overall, this requirement has had a salutary effect on bioethics commissions, since voicing one's views “on the record” has typically made members and witnesses alike attentive to the accuracy, relevance, and comprehensibility of what they say, which has facilitated the eventual translation of those ideas “into recommendations about public policy and practical ethical guidance.”^5^ Any future bioethics commission should use the Internet to provide the public more opportunities not only to follow the commission's work but also to offer their views while the commission is still deliberating. The federal advisory committee process is based on an opportunity for public comment. The Internet has made it possible for a broader segment of the public to offer comments than was the case when the principal means was through attending a commission meeting and speaking during the time devoted to public comments. Such comments can bring in views that may not have been heard from the experts selected to testify, and they can illuminate points in draft reports that need to be clarified, expanded, or even modified.

One characteristic that has been shared by all the bioethics commissions save one has been a strong drive toward consensus. The exception was the President's Council on Bioethics, whose mission reflected the view of its first chair, Leon R. Kass, that “a deep and comprehensive understanding of the issues” was more likely to emerge if it articulated “the complex and often competing moral positions” rather than attempting to reach agreement.^6^ In addition to six reports, the council issued two white papers and two collections that consisted of commissioned papers and classic readings. Yet a well‐crafted report can achieve the goal of fully presenting all opposing viewpoints while also setting forth—and justifying—the conclusions and recommendations on which consensus has been reached. I was repeatedly impressed that even when starting with just a small core of agreement about a topic, the members of the President's Commission were able, through reading, hearing, and debating a variety of positions, to expand substantially the common ground on which they agreed. For example, as we began work on our report on life‐sustaining care, some of the commissioners were of the belief that it was morally and legally wrong for physicians ever to withdraw such care; their views changed as they examined other aspects of care at the end of life, beginning from an agreement that patients should be able to refuse such care when it would not provide an outcome that they valued. A commission's only true power—the power of persuasion—is stronger with unanimity.

**Table:** 

U.S. Federal Bioethics Bodies and Their Major Publications	
Body	Report, white paper, or collection
National Commission for the Protection of Human Subjects of Biomedical and Behavioral Research (1974‐1978; authorized by Congress in 1974; appointed by the Secretary of Health, Education, and Welfare)	*Research on the Fetus* (1975) *Research Involving Prisoners* (1976) *Research Involving Children* (1977) *Psychosurgery* (1977) *Disclosure of Research Information under the Freedom of Information Act* (1977) *Research Involving Those Institutionalized as Mentally Infirm* (1978) *Ethical Guidelines for the Delivery of Health Services by DHEW* (1978) *Institutional Review Boards* (1978) *Special Study: Implications of Advances in Biomedical and Behavioral Researc*h (1978) *The Belmont Report: Ethical Principles and Guidelines for Protection of Human Subjects of Biomedical and Behavioral Research* (1978)
President's Commission for the Study of Ethical Problems in Medicine and Biomedical and Behavioral Research (1980‐1983; authorized by Congress in 1978; initial eleven commissioners appointed by President Carter, with eight vacancies filled by President Reagan)	*Defining Death: Medical, Legal, and Ethical Issues in the Determination of Death* (1981) *Protecting Human Subjects: The Adequacy and Uniformity of Federal Rules and Their Implementation* (1981) *Whistleblowing in Biomedical Research: Policies and Procedures for Responding to Reports of Misconduct* (1981) *IRB Guidebook* (1981) *Compensating for Research Injuries: The Ethical and Social Implications of Programs to Redress Injured Subjects* (1982) *Splicing Life: The Social and Ethical Issues of Genetic Engineering with Human Beings* (1982) *Making Health Care Decisions: The Ethical and Legal Implications of Informed Consent in the Patient‐Practitioner Relationship* (1982) *Deciding to Forego Life‐Sustaining Treatment: Ethical, Medical, and Legal Issues in Treatment Decisions* (1983) *Implementing Human Research Regulations: The Adequacy and Uniformity of Federal Rules and of Their Implementatio*n (1983) *Screening and Counseling for Genetic Conditions: The Ethical, Social, and Legal Implications of Genetic Screening, Counseling, and Education Programs* (1983) *Securing Access to Health Care* (1983) *Summing Up* (1983)
Biomedical Ethics Advisory Committee (1988‐1990; created and appointed by Congress)	None.
Advisory Committee on Human Radiation Experiments (1994‐1995; chartered and appointed by President Clinton)	*The Human Radiation Experiments: Final Report of the President's Advisory Committee* (1995)
National Bioethics Advisory Commission (1996‐2001; chartered and appointed by President Clinton)	*Cloning Human Beings* (1997) *Research Involving Persons with Mental Disorders That May Affect Decisionmaking Capacity* (1998) *Research Involving Human Biological Materials: Ethical Issues and Policy Guidance* (1999) *Ethical Issues in Human Stem Cell Research* (1999) *Ethical and Policy Issues in International Research: Clinical Trials in Developing Countries* (2001) *Ethical and Policy Issues in Research Involving Human Participants* (2001)
President's Council on Bioethics (2001‐2009; chartered and appointed by President George W. Bush)	*Human Cloning and Human Dignity: An Ethical Inquiry* (2002) *Beyond Therapy: Biotechnology and the Pursuit of Happiness* (2003) *Being Human: Readings from the President's Council on Bioethics* (2003) *Monitoring Stem Cell Research* (2004) *Reproduction and Responsibility: The Regulation of New Biotechnologies* (2004) *White Paper: Alternative Sources of Pluripotent Cells* (2005) *Taking Care: Ethical Caregiving in Our Aging Society* (2005) *Human Dignity and Bioethics: Essays Commissioned by the President's Council on Bioethics* (2008) *The Changing Moral Focus of Newborn Screening: An Ethical Analysis by the President's Council on Bioethics* (2008) *Controversies in the Determination of Death: A White Paper* (2008)
Presidential Commission for the Study of Bioethical Issues (2010‐2016; chartered and appointed by President Obama)	*New Directions: The Ethics of Synthetic Biology and Emerging Technologies* (2010) *“Ethically Impossible” STD Research in Guatemala from 1946 to 1948* (2011) *Moral Science: Protecting Participants in Human Subjects Research* (2011) *Privacy and Progress in Whole Genome Sequencing* (2012) *Safeguarding Children: Pediatric Medical Countermeasure Research* (2013) *Anticipate and Communicate: Ethical Management of Incidental and Secondary Findings in the Clinical, Research, and Direct‐to‐Consumer Contexts* (2013) *Gray Matters: Integrative Approaches for Neuroscience, Ethics, and Society* (2014) *Ethics and Ebola: Public Health Planning and Response* (2015) *Gray Matters: Topics at the Intersection of Neuroscience, Ethics, and Society* (2015) *Bioethics for Every Generation: Deliberation and Education in Health, Science, and Technology* (2016)

## What Are the Goals?

A consensus is most easily achieved when a topic is one for which the commission can be a *final stop*. Such topics are of two types: those where many years of debate have produced a general agreement and those that have only recently been identified but that are not controversial. Sometimes, a bioethics commission's report on the first sort of problem will allow activities to go forward with little debate for years, as was the case with the President's Commission report on the determination of death,^7^ but sometimes, a report that restates the consensus among experts in the field does not succeed in producing a change of practice, as was the case with its report on compensating injured research subjects,^8^ a topic on which federal biomedical research offices have been prodded but have failed to act for four decades. (See the table for a list of reports by federal bioethics commissions.)

In addressing a second category of topics—where the commission serves as a *crucible*, “hammering out conclusions on controversial issues when consensus … is not yet apparent”^9^—a commission does its greatest service for the public, which probably explains the very positive reception for the President's Commission's work on decisions about care at the end of life. The topic was then very controversial, but that report was the commission's most widely distributed and most influential with courts and policy‐makers.^10^ Subsequent commissions also took on problems that had not previously been examined yet managed to produce reports that were praised for their clear and comprehensive treatment of difficult topics, as evidenced by the 1995 report of the Advisory Committee on Human Radiation Experiments and the 2011 Presidential Commission for the Study of Bioethical Issues report “*Ethically Impossible”: STD Research in Guatemala*.^11^

A third function of commissions lies at the opposite pole from consensus, namely, serving as a *dumping ground* for an issue that legislative or executive officials have to appear to treat seriously but really want to dispose of. Sometimes the result can be still be felicitous, in which case the commission is really more of a *lightning rod*, absorbing a public policy shock by conducting a study of a sensitive issue in a manner that is less fraught than a congressional hearing or the like and that manages, if not to provide the final word, at least to focus thinking on real issues and to offer sound means of resolving them. For example, in 1980, the White House was under pressure from the country's three major religious organizations (The National Council of Churches, the Synagogue Council of America, and the United States Catholic Conference) to address the “fundamental dangers” of human genetic engineering. At the request of President Carter's science advisor, the President's Commission added this topic to its agenda and produced a report that soothed the more frenzied worriers, critically examined the likely scientific developments, and offered alternative means for continuing oversight within the federal regulatory and funding agencies while also protecting the field from draconian regulations or statutes.^12^

A final function is to investigate the operation of the regulations designed to protect human subjects in research. The 1974 act that created the National Commission specified that after it had recommended new rules for research on human subjects, continuing oversight of the rules’ implementation would be the responsibility of a successor body, the National Council for the Protection of Human Subjects. Once Congress decided to have a body—the President's Commission—with authority beyond that of the Department of HEW, it decided to give the oversight duties to the commission. In this role as *watchdog*, a commission can both throw light on weaknesses in existing regulations or their enforcement and propose a different approach to ensuring that ethical standards are met. That's what the President's Commission did in recommending in 1981 that all federal departments and agencies that conduct or sponsor research on human subjects adopt a single, unified set of rules^13^—a recommendation that was accepted, though it took a decade for the affected federal entities to agree on and issue the Common Rule. Subsequent commissions have not had this function, which is left to advisory bodies within executive departments, such as the Department of Health and Human Services Secretary's Advisory Committee on Human Research Protections, but such boards lack the breadth of authority and independence of a presidentially appointed commission.

## What Should Be Its Status?

Besides independence, the President's Commission had another important, albeit imperfect, power. Called “action‐forcing authority,”^14^ it consisted of being able to issue recommendations to any federal department or agency to take certain steps “with respect to its rules, policies, guidelines, or regulations.” Within sixty days, the agency was required to publish the recommendations in the Federal Register and accept written comments on them from members of the public. Then, the agency was required, within 180 days of the publication, to “provide the Commission with, and publish in the Federal Register, a notice of [its] determination (including an adequate statement of the reasons for the determination)” either to take the recommended steps or explain why such action was inappropriate.^15^ As a practical matter, this authority was less than it seemed, since the only way that a commission could enforce it would be if Congress—which has real power—was unhappy with an agency's failure to respond in a timely fashion. Moreover, once a commission goes out of existence, no one is around to press for action.

These problems should be instructive for anyone intending to create a new commission. First, a bioethics commission should be created by Congress and appointed by the president; its authorizing statute should assign it specific tasks, to which the president or the commission itself should be able to add. That arrangement gives both branches a stake in the commission's work (which is important since many of the most important topics will require legislative as well as executive action), and the link to Congress will give the commission greater power, both formally (as in the case of action‐forcing authority) and informally.

Second, the commission's term should be open‐ended, with limited, staggered terms for members, who should come from the public; any information about governmental activity can come through official liaison officers appointed by the affected agencies. Having continuity over time would allow a commission to build on past successes, even if it works, as its predecessors have, in an inductive, context‐specific fashion rather than by articulating a set of grand principles and rules and then applying them to new problems as they arise. Medicine and research have not finished producing ethical dilemmas, and—as has been true repeatedly over the past forty years—the country would be well served to have a bioethics commission to which the latest problem can be readily referred. In France, the National Consultative Ethics Committee for Health and Life Sciences was established on February 23, 1983, just as the U.S. President's Commission was completing its work. The French committee has functioned well through many administrations, with successive presidents appointing new members as terms expire or vacancies occur. It is available to take up new issues referred to it by the president, the houses of Parliament, government departments, the universities, and even the public; it has issued more than 120 reports on a wide range of topics. Its longevity and ability to rise above politics (which can be quite volatile in France as well) illustrate the value of having an ongoing body, rather than distinct commissions, newly created by successive administrations.
